# F28. PROGRESSIVE POST-ONSET REORGANISATION OF MRI-DERIVED CORTICAL THICKNESS IN ADOLESCENTS WITH SCHIZOPHRENIA

**DOI:** 10.1093/schbul/sby017.559

**Published:** 2018-04-01

**Authors:** Lena Palaniyappan, Anthony James

**Affiliations:** 1 University of Western Ontario; 2 University of Oxford

## Abstract

**Background:**

Cortical thickness changes continuously throughout healthy adolescence reflecting ongoing maturation. In schizophrenia, distributed abnormalities in cortical maturation are suspected. To study if these distributed changes are a result of a co-ordinated process, we investigated the structural covariance among the longitudinal post-onset thickness changes that occur across various brain regions in adolescent-onset schizophrenia.

**Methods:**

19 healthy adolescents and 18 age-matched patients with early-onset schizophrenia were scanned twice (~2 years’ interval). The rate of change in cortical thickness was estimated both at lobar and sulcogyral level. Group level structural covariance was studied using a graph theoretical framework.

**Results:**

At baseline, patients had distributed reduction in cortical thickness compared to controls, though this deviation was abolished over the next 2 years. Occipital cortex had a significantly deviant rate of change in patients (0.8% increase per year) compared to controls (2.5% thinning/year). Patients had a significant increase in covariance of right anterior insula and calcarine sulcus with rest of the brain.

**Discussion:**

Post-onset structural changes in EOS are not a result of random, mutually independent processes. A spatially interconnected reorganization process, distinct from normal maturational events may underlie these distributed changes.

